# Does Speaking Two Dialects in Daily Life Affect Executive Functions? An Event-Related Potential Study

**DOI:** 10.1371/journal.pone.0150492

**Published:** 2016-03-18

**Authors:** Yan Jing Wu, Haoyun Zhang, Taomei Guo

**Affiliations:** 1 Department of Psychology, University of Sheffield, Sheffield, United Kingdom; 2 State Key Laboratory of Cognitive Neuroscience and Learning & IDG/McGovern Institute for Brain Research, Beijing Normal University, Beijing, China; 3 Center for Collaboration and Innovation in Brain and Learning Sciences, Beijing Normal University, Beijing, China; 4 Department of Psychology, Pennsylvania State University, State College, PA, United States of America; University of Western Ontario, CANADA

## Abstract

Whether using two languages enhances executive functions is a matter of debate. Here, we take a novel perspective to examine the bilingual advantage hypothesis by comparing bi-dialect with mono-dialect speakers’ performance on a non-linguistic task that requires executive control. Two groups of native Chinese speakers, one speaking only the standard Chinese Mandarin and the other also speaking the Southern-Min dialect, which differs from the standard Chinese Mandarin primarily in phonology, performed a classic Flanker task. Behavioural results showed no difference between the two groups, but event-related potentials recorded simultaneously revealed a number of differences, including an earlier P2 effect in the bi-dialect as compared to the mono-dialect group, suggesting that the two groups engage different underlying neural processes. Despite differences in the early ERP component, no between-group differences in the magnitude of the Flanker effects, which is an index of conflict resolution, were observed in the N2 component. Therefore, these findings suggest that speaking two dialects of one language does not enhance executive functions. Implications of the current findings for the bilingual advantage hypothesis are discussed.

## Introduction

Bilingual individuals have the ability to speak two languages in everyday life. A major debate in bilingualism research is whether using two languages leads to specific enhancement in non-linguistic cognitive functions, such as executive control (for a review see [1]). On the one hand, studies using the classic Simon task and the Attention Network Test (ANT) have shown that bilinguals are better at ignoring interference from irrelevant stimuli [2–8]. Prior & Macwhinney [9] examined a group of English monolinguals and a group of English speakers who also speak a second language (i.e., English-X bilinguals) using a task-switching paradigm that did not involve language processing. The two groups of participants were matched on a selection of non-linguistic variables that might affect their performance in the task (e.g., attentional span and short-term memory). Results suggested that, as compared to the monolingual participants, the bilingual participants suffered less switch cost, indicating a better ability to switch between tasks, evidence that is consistent with the bilingual advantage hypothesis. On the other hand, some recent studies have failed to find the bilingual advantage effect [10–15] and other studies have suggested that the observation of bilingual advantage effects is due to the difference in background factors such as education level and socioeconomic status (SES) between the monolingual and bilingual participants[16, 17].). For example, in a comprehensive investigation, Paap and Greenberg [11]compared bilingual with monolingual participants in antisaccade, Simon, flanker, and colour-shape switching tasks. The results showed that, among 15 indicators of executive processing, between-group difference was only found in one indicator, and the direction of this difference suggested a disadvantage in bilinguals.

To maintain smooth verbal communications, bilinguals need to switch between two languages and inhibit interference from the unintended language to the target language [18–21]. These experiences, according to the theory of neuroplasticity[22], may enhance the functional level of neural mechanisms that are involved in task-switching and inhibition control, leading to superior performance in relevant non-linguistic tasks. This explanation is supported by a series of neuroimaging studies showing that performing non-linguistic tasks that require inhibition control activates a wider neural network in bilingual as compared to monolingual individuals [23, 24]. Moreover, as compared to monolinguals, bilinguals engaged in non-linguistic tasks demonstrated stronger neural activations in brain areas typically associated with language control such as the DLPFC and ACC [25–27], suggesting that bilinguals recruit additional neural resources to maintain executive functions with an enhanced efficiency. However, some of these studies have failed to report behavioural results that match the observed differences in neural mechanisms [23, 24] Therefore, it is important to keep in mind the difficulties and inconsistency when interpreting neuroanatomical evidence for cognitive advantage in bilinguals [28].

Human language is a complicated system containing a number of subsystems including phonology, orthography, grammar, syntax, morphology, and semantics. One novel perspective from which to study the effect of language on domain-general cognitive functions is to look at how the experience of controlling one, several, or all subsystems between two languages affects non-linguistic performance. This is an important question since the answer will further specify the nature of the interaction between language and cognition at the level of language subsystem. Previous studies comparing monolingual and bilingual speakers, who use two completely independent language systems, cannot tease apart the role of different language domains.

To examine the relative contributions of language subsystems to cognitive functions, for the first time we compared two groups of Chinese native speakers, one of which uses only the standard Chinese Mandarin (i.e., Pu Tong Hua) and the other uses both the standard Chinese Mandarin and the Southern Min (e.g., the Min Nan) dialect. Chinese has one of the most diversified and complicated dialect distributions. Some dialects in south China, for example the Southern Min dialect, the Hakka dialect, and the Wu dialect, have phonological variations (e.g., Tone sandhi) so distinct from the standard Chinese Mandarin and other Chinese dialects that speakers of different dialects cannot understand one another verbally. Interestingly, except for phonology, these dialects share all other subsystems including orthography with the standard Chinese Mandarin [29–31]. Therefore, Chinese dialect speakers form the most ideal context in which the specific effect of language subsystems on non-linguistic performance can be examined. We applied behavioural and electrophysiological measurements to investigate the potential effect of speaking an additional dialect on the performance of the classic Flanker task [32, 33]. Event-related potentials (ERPs) are brain activities recorded continuously from the participant’s scalp, time-locked to neural operations under investigation. The high temporal resolution of ERPs is particularly effective in the study of language processing, as it unfolds rapidly. However, to date, very few studies have applied ERPs in the study of neural processes underlying bilinguals’ and monolinguals’ cognitive performance (but see [34]). Our hypothesis is that if the experience of using two phonological systems (rather than two entirely different languages) on a regular basis affects executive functions, mono-dialect and bi-dialect speakers should have different performance in the Flanker task: bi-dialect speakers should show a smaller Flanker effect, in the form of reduced reaction times and error rates as well as associated ERP variations, as compared to mono-dialect speakers. The Flanker effect measures the interference cost by comparing performance in the congruent and the incongruent trials. A reduced Flanker effect in the bi-dialect speakers would suggest enhanced ability of ignoring irrelevant stimuli and/or screening out distracters in this group of participants, evidence that is consistent with the bilingual advantage hypothesis. The absence of such a between-group difference would fail to support this hypothesis.

## Methods

### Ethics Statement

This study was approved by the Institutional Review Board of the Imaging Center for Brain Research of Beijing Normal University. All participants gave written informed consent prior to the experiment and were paid for their participation.

### Participants

**Fig 1:**
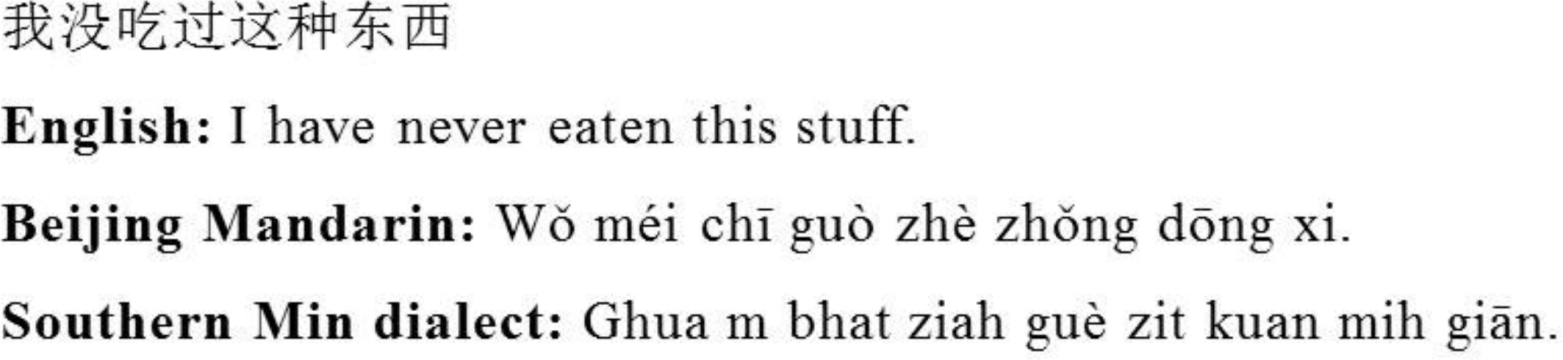
Examples of the standard Chinese Mandarin and Southern Min Nan dialect. As can be seen in the PinYin (phonetic transcription of Chinese characters), the two dialects have distinct pronunciations. Other language domains such as orthography, syntax, semantics (e.g., meaning of individual characters), and grammar are comparable.

Forty-eight native Chinese speakers who had normal or corrected-to-normal vision and no self-reported neurological disorders participated in the experiment. Half of them speak only Standard Mandarin (The mono-dialect group; Mean age = 20.90; SD = 2.22). The other half speak the Standard Mandarin and another main Chinese dialect such as the Southern Min Nan dialect or the Hakka dialect (The bi-dialect group; Mean age = 20.37; SD = 2.06). All the dialects have distinct phonological systems from that of the Standard Mandarin (see Fig 1 for an example), resulting in opacity in verbal communication. However, the dialects and the Standard Mandarin are highly comparable in terms of orthography, semantics, grammar, and syntax (There are small differences in oral vocabulary and syntax between standard Chinese Mandarin and some southern dialects [35, 36]). After excluding data of low EEG quality, 20 mono-dialect and 19 bi-dialect participants were included in the final analysis. In a language history questionnaire, the bi-dialect speakers rated the dialect as more proficient than the Standard Mandarin (*t* (18) = 2.43, *p* = 0.026). Between-group comparisons showed that the English proficiency between the two groups was not different (*p* > .01), but the Standard Mandarin proficiency of the bi-dialect group was rated lower than that of the mono-dialect group (*t* (37) = 2.83, *p* = 0.007). However, the dialect proficiency of the bi-dialect group was comparable with the Mandarin proficiency of the mono-dialect group (*p* > .1). The two groups were matched on factors that may influence executive functions. These factors include IQ (measured by Raven Progressive Matrices [37]), working memory (measured by the Operational Span Task [38]), and demographic variables (e.g., age, gender, education background, Socioeconomic Status; *p*s > .1 in all comparisons; See Table 1).

**Table 1:** *Demographic variables*, *language proficiencies*, *and other general cognitive abilities of the mono-dialect and bi-dialect group*.

	Mono-dialect	Bi-dialect
Age	20.90 (2.22)	20.37 (2.06)
Gender (male/female)	3/17	6/13
Standard Chinese Mandarin proficiency	9.62 (0.58)	8.94 (0.88)
Dialect proficiency		9.63 (0.76)
English proficiency	5.96 (1.40)	6.09 (1.13)
Raven score	57.60 (2.21)	56.37 (2.87)
Operational span recall	51.95 (5.62)	48.89 (7.08)
Education background	3.15 (0.37)	3.05 (0.62)
Family income	3.00 (0.86)	3.00 (1.15)

Note: Ratings are presented for each group of participants and standard deviations are given in parentheses. Participants’ dialect and language proficiency were self-rated on a scale between 1 (lowest) and 10 (highest). All students in China study English in the classroom from secondary school, but none of the participants use English in everyday life or have experience of living in an English-speaking society. Their working memory and non-verbal intelligence were measured by using the Operational span recall (60 in total) and Raven test (60 in total). Participants also rated their education level and family income on a 1 to 5 and a 1 to 4 scale, respectively, with 1 as the lowest rating and 5 or 4 the highest.

### Materials and procedure

**Fig 2:**
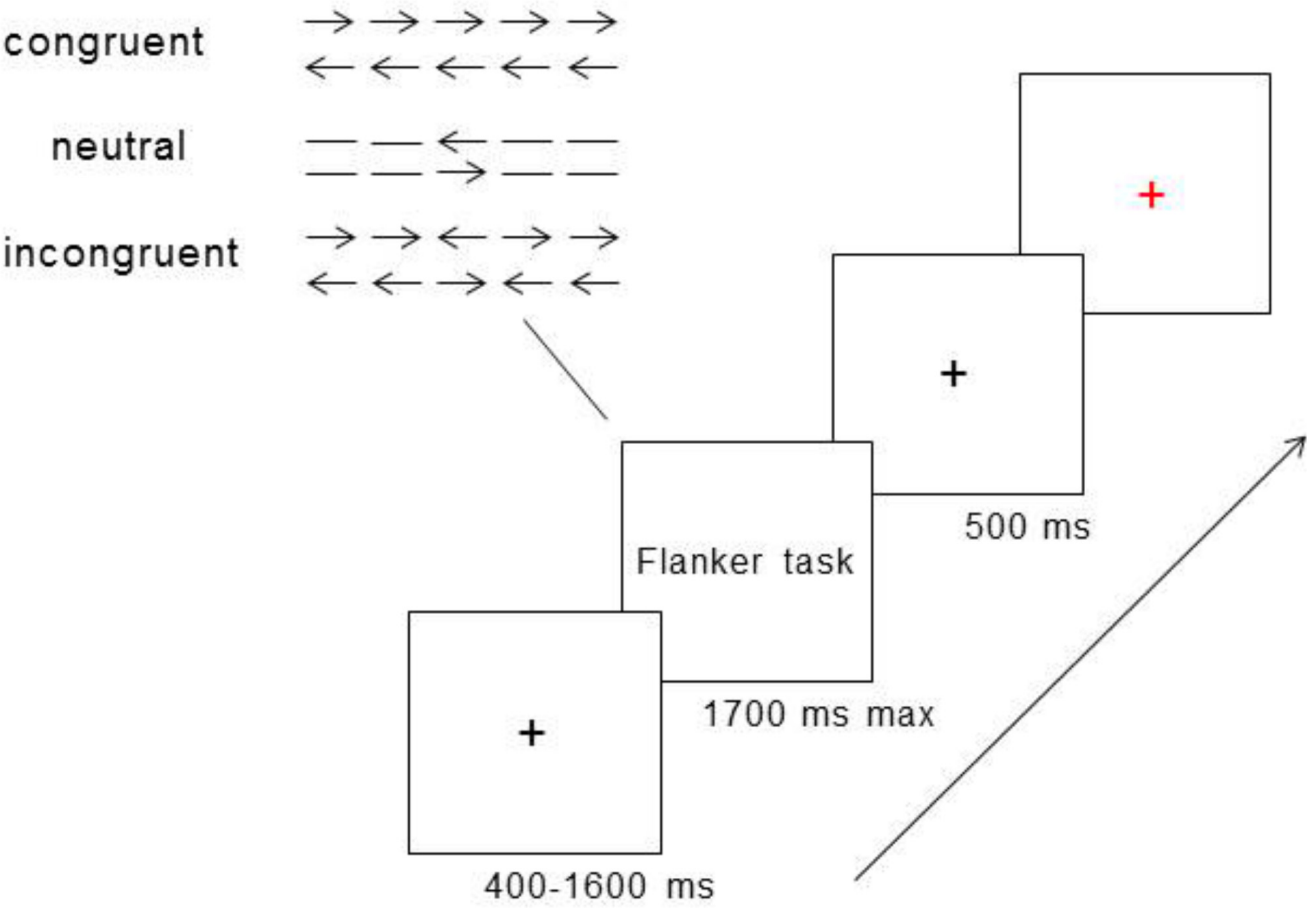
A flow chart of the Flanker task and examples of the stimuli. Each experimental trial was 4 seconds in duration including, in a sequential order, a pre-stimuli fixation cross (black, 400–1600 ms), the Flanker task (maximum of 1700 ms or as soon as the participant made a response), a post-stimuli fixation cross (black, 500 ms), and an inter-trial interval fixation cross (red, varied length).

In the classic Flanker task [33], the stimuli consisted of five horizontal arrows or lines. Participants were required to indicate the direction of the central arrow (i.e., the target) while ignoring the peripheral arrows (i.e., the flankers) by pressing buttons. In the congruent and incongruent condition, the directions of target and peripheral arrows were identical and opposite, respectively. In the neutral condition, the flankers consisted of lines instead of arrows (i.e., showing no preferences to directions). Each trial began with a black fixation cross presented for 400–1600 ms on a white background, and then the flanker task was displayed until the participant responded to it or for a maximum duration of 1700 ms. The trial ended with a black cross displayed for 500 ms and inter-stimuli intervals were occupied by a red fixation cross, indicating to the participants that it was fine to blink (See Fig 2). The red fixation cross was presented for an unfixed period of time so that each experimental trial was 4 seconds long. Twelve practice trials were presented before the start of the formal experiment, which consisted of 6 blocks of 48 trials (i.e., 16 trials for each of the 3 conditions).

### EEG acquisition and analysis

Electrophysiological data were recorded in reference to the right mastoid at a rate of 1 kHz from 32 Ag/AgCl electrodes (Neuroscan Inc.) placed according to the extended 10–20 convention. Impedances were kept below 5kΩ.

Electroencephalogram (EEG) was bandpass filtered on-line between 0.05 and 100 Hz and refiltered off-line with a 30 Hz, low-pass, 24 dB/oct, zero-phase shift digital filter. Eye blinks were mathematically corrected [39], and remaining artifacts were manually dismissed. Epochs ranged from 100ms before stimulus onset to 600 ms after stimulus onset of the Flanker task. Baseline correction was performed in reference to 100 ms pre-stimulus activity and individual averages were re-referenced to linked mastoids. ERP data were artifact-rejected beyond ±70uV. The minimal trials accepted per condition were 40 and nine participants were excluded due to excessive artifact or low data quality. ERP data were collected simultaneously to behavioural data.

The temporal windows of three critical components (P2, N2, and a late positive component) were defined a priori [40, 41] and checked against the Global Field Power measured across the scalp [42]. Mean ERP amplitude at 190-220ms and 160-190ms (FZ, FCZ, F3/4, FC3/4) for the mono-dialect and the bi-dialect participants, respectively, 300-350ms (FZ, FCZ, F3/4, FC3/4), and 370-420ms (FZ, FCZ, CZ, F3/4, FC3/4, C3/4) were subjected to a repeated-measures ANOVA with Flanker task (congruent, incongruent, and neutral trials) as the within-subject factor, and group (mono-dialect and bi-dialect speakers) as the between-subject factor. Mean peak latencies from electrodes (FZ, FCZ, F3/4, FC3/4) between 160 and 220ms, a temporal window that covers the P2 components in both groups, were subjected to a repeated-measure ANOVA with Flanker task (congruent, incongruent, and neutral trials) as the within-subject factor, and group (mono-dialect and bi-dialect speakers) as the between-subject factor. Greenhouse–Geisser correction was used where applicable.

## Results

### Behavioural data

**Fig 3:**
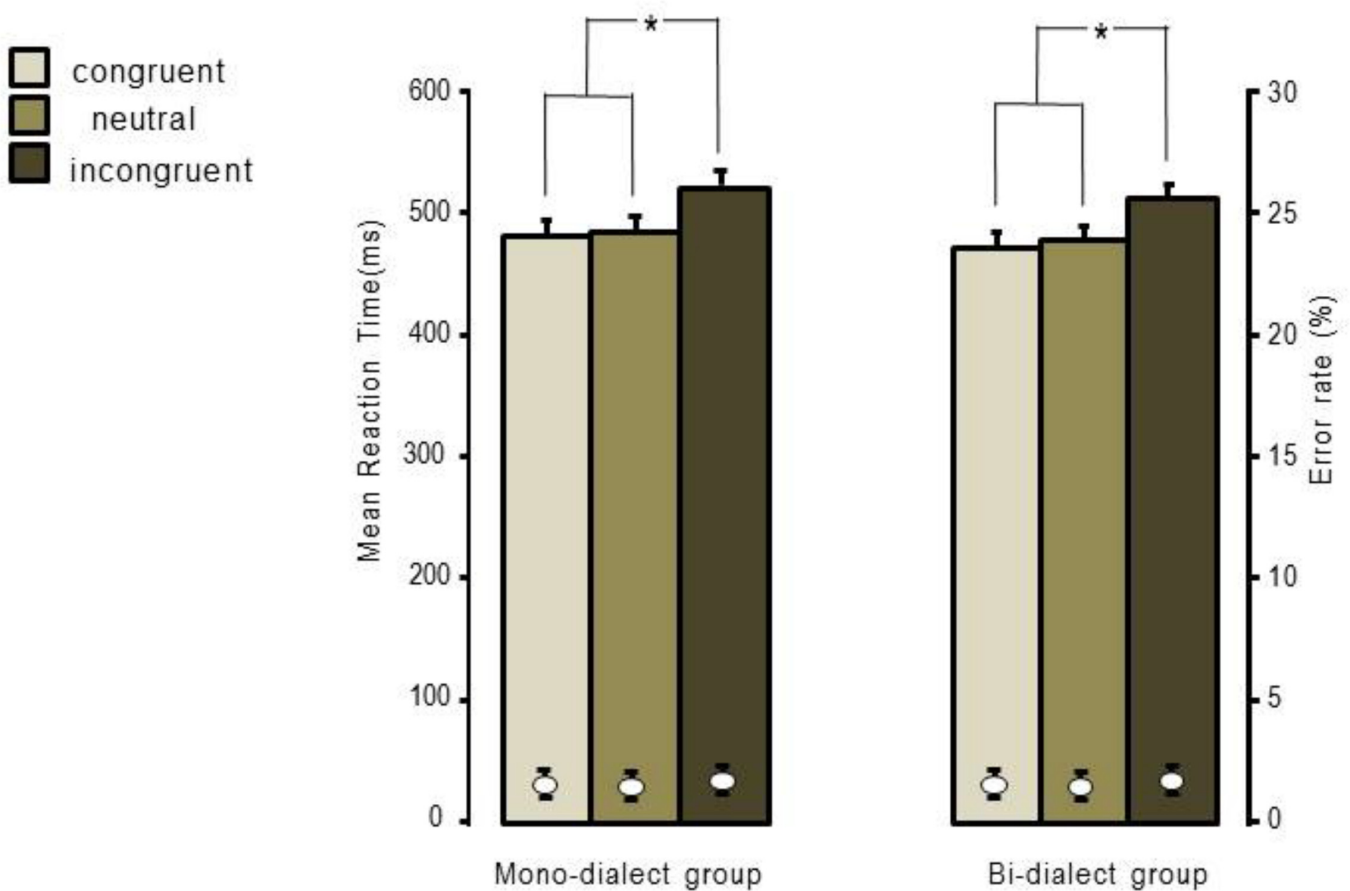
Behavioural results of the Flanker task in the mono-dialect and bi-dialect group. Bars represent the reaction times in ms (to the right-hand y axis) and bullet points represent error rates in percentage (to the left-hand y axis). Error bars show the standard errors. Stars indicate where significant difference was found.

Only trials with correct responses made between 300 and 1400 ms and trials between mean reaction times ±2.5 standard deviations were included in the analysis. This resulted in the exclusion of 2.8% of total trials at the analysis. A series of repeated measures ANOVAs with language as the between-subject factor (mono-dialect and bi-dialect group) and Flanker conditions as the with-subject factor (congruent, incongruent, and neutral) were performed on both RTs and error rate (See Fig 3). Analyses of RT showed a main effect of Flanker conditions (*F* (2, 74) = 143.24, *p* < 0.001), no significant effect of group (F (1, 37) = 0.23, *p* > 0.1), and no significant interaction between the two factors (F (2, 74) = 0.19, *p* > 0.1). Post-hoc analysis (i.e., paired sample t-test) confirmed that the RT of incongruent trials was significantly longer than that of congruent trials (*p* < 0.001) and neutral trials (*p* < 0.001). In the analyses of error rate, the main effect of Flankers was significant (*F* (2, 74) = 7.05, *p* < 0.005), but group difference (F (1, 37) = 0.54, *p* > 0.1) and interaction (F (2, 74) = 0.68, *p* > 0.1) were both non-significant. Post-hoc analysis showed that the error rate of incongruent trials was higher than that of congruent trials (*p* < 0.05) and neutral trials (*p* < 0.05).

### ERP data

Repeated measures ANOVAs conducted on the peak latencies of the P2 component revealed a significant main effect of group (*F* (1, 37) = 4.38, *p* < 0.05), showing that the P2 was found earlier in the bi-dialect (M = 189.72, SD = 18.95) as compared to the mono-dialect group (M = 201.39, SD = 17.75). The analysis also showed a main effect of the Flanker task, (*F* (2, 74) = 11.54, *p* < 0.001) and a significant interaction between group and Flanker task, (*F* (2, 74) = 4.73, *p* < 0.05). Further analysis showed that there was a significant main effect of the Flanker task in the bi-dialect group (*F* (2, 38) = 8.77, *p* < 0.05), but not in the mono-dialect group (*F* (2, 38) = 3.18, *p* = 0.06). In the bi-dialect group, pairwise comparisons showed significant differences in the P2 latency between the neutral condition (M = 196.75, SD = 19.73) and the congruent condition (*p* < .05; M = 185.36, SD = 18.04), and also between the neutral condition and the incongruent condition (*p* < .05; M = 187.06, SD = 17.97).

Repeated measures ANOVAs conducted on the mean amplitude of the P2 revealed a significant main effect of the Flanker task (*F* (2, 74) = 8.03, *p* < 0.001), no main effect of group (*F* (1, 37) = 1.83, *p* < 1), and a significant interaction between group and the Flanker task (*F* (2, 74) = 3.84, *p* < 0.05). Post-hoc analysis revealed a significant group difference in the processing of the neutral stimuli, (independent-sample t-test; *t* (37) = 2.20, *p* < 0.05), featuring a reduced P2 in the bi-dialect as compared to the mono-dialect group. Paired-sample t-tests showed that, for the bi-dialect participants, the amplitude in the neutral condition is significantly lower than that in the congruent and the incongruent condition (*p*s < 0.05).

A main effect of the Flanker task was found on mean amplitude analysis of the N2 component (*F* (2, 74) = 10.13, *p* < 0.001). Pairwise comparison showed that N2 was significantly larger (i.e. more negative) in the incongruent as compared to the congruent and neutral conditions (*p*s < 0.005). Group difference (*F* (1, 37) < 1) and interaction (*F* (2, 74) < 1) were both not significant.

**Fig 4:**
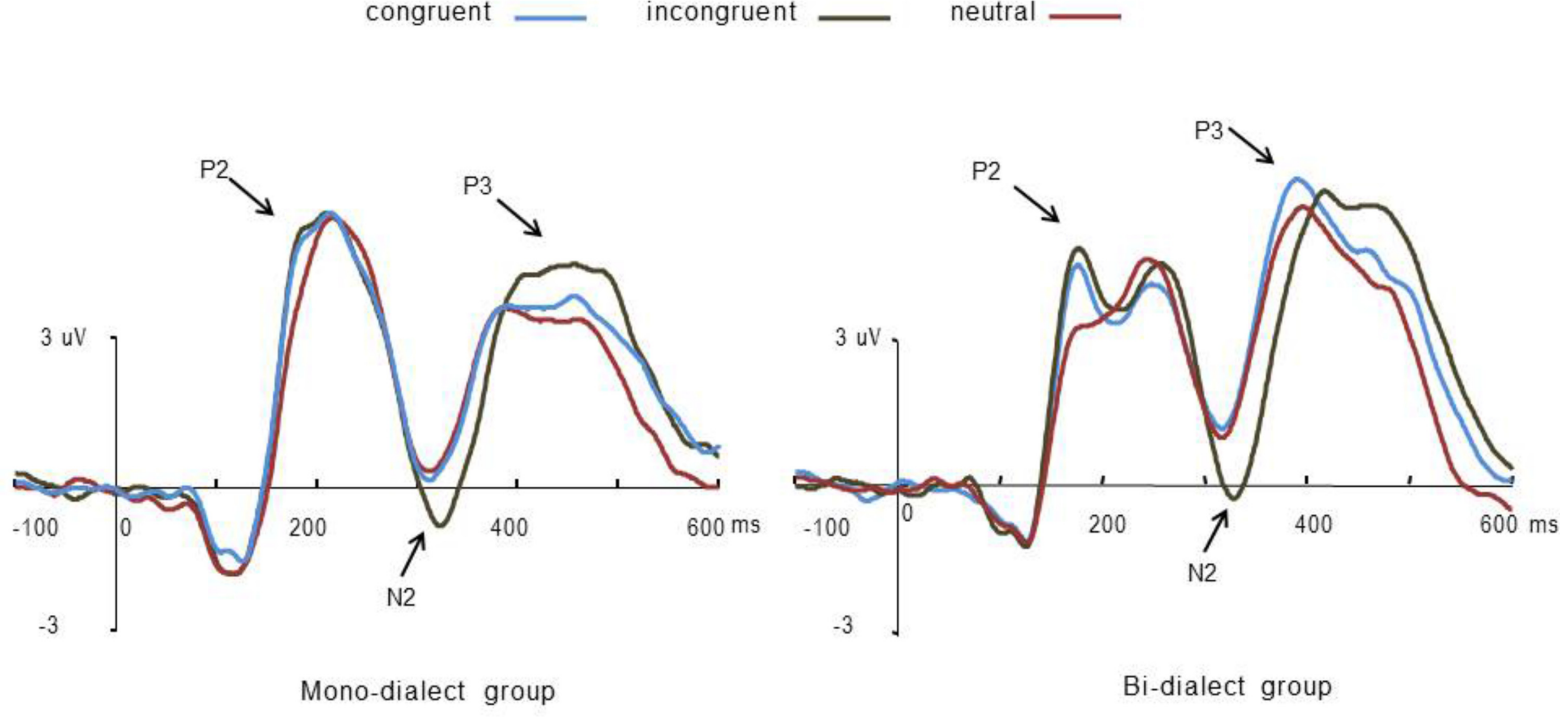
ERPs elicited by the Flanker task in the mono-dialect and bi-dialect group. Averaged ERPs from six frontal electrodes FZ FCZ F3/4 FC3/4 are presented for the congruent (light blue), incongruent (brown), and neutral (dark red) condition. Arrows indicate the ERP component on which statistical analysis was carried out.

At the late positive component, a significant main effect of group on mean amplitude was observed (*F* (1, 37) = 4.04, *p* < 0.05). The main effect of the Flanker task was not significant (*F* (2, 74) = 1.10, *p* = 0.33), and the interaction of the Flanker task and group was also not significant (*F* (2, 74) = 2.48, *p* = 0.09). Post-hoc analysis showed that, in the congruent condition, a larger amplitude modulation was elicited in the bi-dialect as compared to the mono-dialect group (*t* (37) = 2.87, *p* < 0.05). In the neutral condition, between-group comparison showed a marginal significant difference toward the same direction as that of the congruent condition (*t* (37) = 1.98, *p* = 0.055). In the incongruent condition, the two groups were not significantly different (t (37) = 1.12, p = 0.27; See Fig 4).

## Discussion

The current study compared executive functions of native Chinese speakers who speak only the Standard Chinese Mandarin with those who speak both the Standard Chinese Mandarin and a phonologically distinguished Chinese dialect. Unlike other experimental paradigms such as the Stroop task, the Flanker task used in the current study is highly non-linguistic because the task demand draws upon natural visual-motor correspondences rather than an artificial association which may elicit sub-vocalization. The two groups of participants were well-matched on a selection of variables, minimizing the possibility that between-group comparisons are influenced by extra-linguistic differences [10]. Behavioural results showed the classical Flanker effect (e.g.,[43]) in both groups. However, inconsistent with the cognitive advantage hypothesis, our findings showed no significant difference in either reaction times or error rates between the mono-dialect and the bi-dialect group. One possibility is that the two groups of participants are in theory bilingual individuals, as college students in China must learn English as their second language from junior high school. The knowledge in a second language might have attenuated the potential difference between mono-dialect and bi-dialect speakers in executive functions. However, since participants do not use English on a regular basis and are matched for English proficiency (see Table 1), these findings are more in line with the following explanation: speaking two dialects does not lead to behavioural changes in executive function tasks.

Interestingly, analysis of the ERP data revealed differences in several time courses between the two groups of participants. The P2 component was observed earlier in the bi-dialect group which peaked between 160 and 190 ms as compared to the mono-dialect group which peaked between 190 and 220 ms. Since the P2 has been associated with the attentional orientation [44–46], an earlier P2 suggests that bi-dialect participants were quicker at attending to the Flanker task as compared to mono-dialect participants. However, analysis of the ERP mean amplitude at the P2 component showed that no Flanker effect (e.g., differences between the congruent and incongruent conditions) was found in either the mono-dialect or the bi-dialect group in this particular time window. Therefore, the between-group difference in P2 latency does not suggest that the bi-dialect speakers have an advantage in executive functions as compared to the mono-dialect speakers, because the lack of a Flanker effect suggests that ERP modulations in this time window are not associated with executive functions. The P2 component in the bi-dialect group is more pronounced for the congruent and incongruent conditions as compared to the neutral condition. One explanation is that congruent and incongruent trials have higher visual complexity as compared to neutral trials, leading to increased demand for early, pre-attentive visual processing.

The Flanker effect was found between 300 and 350 ms where, in both groups, the processing of incongruent trials increased the mean ERP amplitude as compared to the neutral and congruent trials, a pattern that is consistent with previous studies using the same task [40, 47, 48]. No between-group difference was found in this time course in any condition. In the subsequent positive component, between-group difference was found in the congruent and neutral conditions, featuring increased mean ERP amplitude in the bi-dialect as compared to the mono-dialect speakers. As no effect was found in the critical incongruent condition, the between-group difference might be related to background differences that do not directly intervene with the performance of the Flanker task.

The present study provides the first ERP evidence for the differences in neural processes underlying executive functions in mono-dialect and bi-dialect speakers. Despite the lack of behavioural effects, bi-dialect speakers showed an earlier neural response to attentional deployment, which is an observation consistent with previous studies that have compared bilinguals and monolinguals [5, 49]. However, there is no evidence for an advantage in conflict resolution in the bi-dialect as compared to the mono-dialect group. One explanation for the pattern of results lies in the unique language experiences of the bi-dialect speakers, who have to rely exclusively on the phonology to dissociate one Chinese dialect from another. Bi-dialect speakers have to quickly attend to subtle changes in pronunciation to maintain a smooth and efficient conversation. However, they do not have to plan sentences according to two sets of rules and switch between them, because the grammar and syntax of the two dialects are highly comparable. Neither do they have to conceptualize abstract meanings from two independent semantic representations, as lexical entities are shared between dialects except for the phonological forms. The lack of a more demanding control experience may explain the absence of a possible advantage in bilinguals. Another possible explanation is that, as pointed out by Morton and Harper [16, 17], bilingual and monolingual speakers often differ in background factors such as the SES, because bilingual individuals tend to come from middle class families whereas the SES background of monolingual individuals is more mixed. This difference in the SES contributed to the bilingual advantage effects that were reported previously. In comparison, bi-dialect and mono-dialect control participants were fully matched on non-linguistic variables, as shown in Table 1, in the current study. The matching procedure eliminated possible between-group differences due to confounding variables.

In conclusion, the experience of controlling two phonological systems (i.e., bidialectualism) on a daily basis does not enhance behavioural performance in an executive function task (i.e., the Flanker task), but it yields generic differences in underlying neural processes associated with the Flanker task between bi-dialect and mono-dialect speakers. Future studies will have to look at individuals with greater differences in language experiences beyond the monolingual-bilingual comparison. These individuals will include bilingual speakers of two distinct languages, bilingual speakers with two overlapping languages, bi-dialect monolingual speakers, and mono-dialect monolingual speakers, and they must be matched on non-linguistic variables that could potentially affect cognitive performance. Such studies will not only examine the validity of bilingual advantage effects that have been reported so far, but may also reveal the particular language experiences as the underlying causes of the effect.

## Supporting Information

S1 Data Minimal Dataset. (ZIP)

S1 Questionnaire Personal Background & SES Questionnaire. (DOCX)

S2 Questionnaire Language History Questionnaire. (DOCX)
