# Supplementary material for: Does Speaking Two Dialects in Daily Life Affect Executive Functions? An Event-Related Potential Study
Source: PLoS One. 2016 Mar 18;11(3):e0150492. doi: 10.1371/journal.pone.0150492 (PMC4798723; doi:10.1371/journal.pone.0150492)
Supplement: S1 Questionnaire — (DOCX) [file pone.0150492.s002.docx]

Supporting information

Personal Background & SES Questionnaire

*This questionnaire is designed to give us a better understanding of your experience. We ask that you be as accurate as thorough as possible when answering the following questions.*

1. Age

2. Gender

3. Education Background

(1) High-school degree (2) Junior college

(3) Bachelor’s degree (4) Master's degree or doctorate

4. Hometown City, Province

5. How often do you operate on the computer or play computer games?

(1) usually (2) often (3) sometimes (4) seldom (5) never

6. How often do you play the piano or other percussion?

(1) usually (2) often (3) sometimes (4) seldom (5) never

7. The average monthly income of your family

(1) <1000 RMB (2) 1000-2000 RMB (3) 2000-3000 RMB (4) >3000 RMB
