# Supplementary material for: Does Speaking Two Dialects in Daily Life Affect Executive Functions? An Event-Related Potential Study
Source: PLoS One. 2016 Mar 18;11(3):e0150492. doi: 10.1371/journal.pone.0150492 (PMC4798723; doi:10.1371/journal.pone.0150492)
Supplement: S2 Questionnaire — (DOCX) [file pone.0150492.s003.docx]

Language History Questionnaire

*This questionnaire is designed to give us a better understanding of your experience on languages. We ask that you be as accurate as thorough as possible when answering the following questions.*

1. Dialect spoken at home:

2. Language used in Elementary education:

3. Describe your language learning and usage.

| Language | Age of Acquisition | Acquisition Background | Use Frequency | Others |
| --- | --- | --- | --- | --- |
|  |  |  |  |  |
|  |  |  |  |  |
|  |  |  |  |  |
|  |  |  |  |  |

4. Please rate your Chinese Mandarin proficiency. (1=not literate and 10=very literate)

Listening Speaking Reading Writing

5. Please rate your Dialect proficiency. (1=not literate and 10=very literate)

Listening Speaking

6. Please rate your English proficiency. (1=not literate and 10=very literate)

Listening Speaking Reading Writing

7. Please rate your proficiency of other languages (If have). (1=not literate and 10=very literate)

Listening Speaking Reading Writing

8. Have you studied / lived abroad?

- Yes
- No

If Yes, where and when did you study, for how long, and what language did you speak?

| Country | Approx. dates | Length of Stay | Language |
| --- | --- | --- | --- |
|  |  |  |  |
|  |  |  |  |

9. Please provide any certification that can prove your language proficiency (CET, TOFEL, IELTS et al.).

*Thank you for your participation!*
